# How multiple episodes of exclusive breastfeeding impact estimates of exclusive breastfeeding duration: report from the eight‐site MAL‐ED birth cohort study

**DOI:** 10.1111/mcn.12352

**Published:** 2016-08-08

**Authors:** Ramya Ambikapathi, Margaret N. Kosek, Gwenyth O. Lee, Cloupas Mahopo, Crystal L. Patil, Bruna L. Maciel, Ali Turab, M Munirul Islam, Manjeswori Ulak, Anuradha Bose, Maribel Paredes Olortegui, Laura L. Pendergast, Laura E. Murray‐Kolb, Dennis Lang, Benjamin J. J. McCormick, Laura E. Caulfield

**Affiliations:** ^1^ Fogarty International Center National Institutes of Health Bethesda Maryland USA; ^2^ Center for Human Nutrition, Department of International Health The Johns Hopkins Bloomberg School of Public Health Baltimore Maryland USA; ^3^ Department of Nutrition, School of Health Sciences University of Venda Thohoyandou Limpopo Province South Africa; ^4^ Department of Women, Children and Family Health Science, College of Nursing University of Illinois at Chicago Chicago Illinois USA; ^5^ Department of Nutrition State University of Ceará Fortaleza Ceará Brazil; ^6^ Department of Paediatrics and Child Health Aga Khan University Karachi Pakistan; ^7^ Centre for Nutrition and Food Security International Centre for Diarrhoeal Disease Research Dhaka Bangladesh; ^8^ Department of Child Health and Institute of Medicine Tribhuvan University Kathmandu Nepal; ^9^ Christian Medical College Vellore India; ^10^ Biomedical Investigations Unit AB PRISMA Iquitos Peru; ^11^ School Psychology Program Temple University Philadelphia Pennsylvania USA; ^12^ Department of Nutritional Sciences The Pennsylvania State University State College Pennsylvania USA

**Keywords:** exclusive breastfeeding, duration, DHS, prevalence, metrics, MAL‐ED, Nepal, Bangladesh, Pakistan, India, Brazil, Peru, Tanzania, South Africa

## Abstract

The duration of exclusive breastfeeding (EBF) is often defined as the time from birth to the first non‐breast milk food/liquid fed (EBFLONG), or it is estimated by calculating the proportion of women at a given infant age who EBF in the previous 24 h (EBFDHS). Others have measured the total days or personal prevalence of EBF (EBFPREV), recognizing that although non‐EBF days may occur, EBF can be re‐initiated for extended periods. We compared breastfeeding metrics in the MAL‐ED study; infants' breastfeeding trajectories were characterized from enrollment (median 7 days, IQR: 4, 12) to 180 days at eight sites. During twice‐weekly surveillance, caretakers were queried about infant feeding the prior day. Overall, 101 833 visits and 356 764 child days of data were collected from 1957 infants. Median duration of EBFLONG was 33 days (95% CI: 32–36), compared to 49 days based on the EBFDHS. Median EBFPREV was 66 days (95% CI: 62–70). Differences were because of the return to EBF after a non‐EBF period. The median number of returns to EBF was 2 (IQR: 1, 3). When mothers re‐initiated EBF (second episode), infants gained an additional 18.8 days (SD: 25.1) of EBF, and gained 13.7 days (SD: 18.1) (third episode). In settings where women report short gaps in EBF, programmes should work with women to return to EBF. Interventions could positively influence the duration of these additional periods of EBF and their quantification should be considered in impact evaluation studies. © 2016 John Wiley & Sons Ltd

## Introduction

Exclusive breastfeeding (EBF) for the first six months of a child's life is a WHO recommended standard and is based on evidence of protection against respiratory infection, diarrheal illness, eczema and allergies in children while providing the optimal nutrition for their growth and development (Kramer & Kakuma 2012). The estimated risk of mortality and morbidity because of lack of EBF in the first six months of life shows a striking dose response effect, and is estimated to be responsible for 823, 000 deaths a year among children under five years of age (Black *et al*. 2008; Victora *et al*. 2016).

Despite this, in developing countries, recommended breastfeeding practices are often not followed for a variety of socio‐cultural and economic reasons (Marquis *et al*. 1997; Cohen *et al*. 1999; Colin & Scott 2002; Dearden *et al*. 2002; Kakute *et al*. 2005; Otoo *et al*. 2009; Senarath *et al*. 2011; Lamberti *et al*. 2011; Badham 2012). A recent study examining global trends in EBF (for the duration of six months) based on Multiple Indicator Cluster Survey and Demographic and Health Survey data from 66 developing countries, found only a marginal increase from 33 to 39% from 1995 to 2010 (Cai *et al*. 2012). In spite of scientific consensus, and increasing awareness of the need to move the EBF recommendation into effective practice, there has been poor progress to date in both developed and developing countries (Morrow & Lutter 2012; World Health Organization (WHO) 2014).

Various indicators have been used to assess EBF duration in the first six months. Because of the difficulty in conducting large longitudinal studies to estimate EBF duration, cross‐sectional surveys in which mothers of young infants are queried regarding EBF in the last 24 h have been used to extrapolate median EBF duration (World Health Organization (WHO) 2003). Several studies have characterized large discrepancies between this method of assessment and longitudinal monitoring methods (Cattaneo *et al*. 2000; Aarts *et al*. 2000; Arifeen *et al*. 2001; Bland *et al*. 2003; Gillespie *et al*. 2006; Agampodi *et al*. 2009; Flaherman *et al*. 2011). For example, Aarts *et al.* reported differences of more than 40% between cross‐sectional vs. longitudinal estimates of EBF prevalence in the first four months among Swedish mother–child dyads (Aarts *et al*. 2000). At six months of age, the absolute difference in EBF prevalence between the methods was nine percentage points (11 vs. 1.8%) (Aarts *et al*. 2000). Recently, Pullum described differences in EBF estimates that result from DHS data when using a prevalence measure vs. interpolation using median duration (Pullum 2014). In 2007, a consensus panel chose the percentage of infants 0–5.9 months reported as EBF the day before as the WHO core indicator to monitor progress in EBF duration. At that time it was recognized that this indicator would overestimate the proportion of infants exclusively breastfed to 6 months, with the degree of positive bias varying depending on the underlying infant feeding pattern in the population (World Health Organization (WHO) 2008).

The Etiology, Risk Factors and Interactions of Enteric Infections and Malnutrition and the Consequences for Child Health and Development (MAL‐ED) is a birth cohort study with intensive follow‐up of infant feeding practices, which allows for a comparison of multiple breastfeeding metrics. The aims of this paper are: (1) to compare multiple EBF indicators and (2) to evaluate maternal and child characteristics for identified differences in these metrics across eight geographically and culturally distinct populations.

**Key messages**

- Differences in metrics used to capture the duration of exclusive breastfeeding may lead to inconsistent results across studies and over time.
- Programme planners should be aware that reported change to predominant and/or partial breastfeeding may not reflect a permanent end to exclusive breastfeeding. Mothers may need appropriate support for increasing their milk supply depending on the switch and the duration of the gap.
- Mothers who report switching away from exclusive breastfeeding early are more likely to continue EBF during the first 6 months of life.

## Methods

The eight sites in the MAL‐ED birth cohort study followed a harmonized protocol to collect information on nutrition, morbidity, gut function, growth, vaccine response and cognitive development for the first two years of the child's life (MAL‐ED Network Investigators 2014). Each site participating in the MAL‐ED network obtained the ethical approval for the study from their respective institutions and written informed consent was obtained from the participants. Throughout the report, the MAL‐ED study sites are referred to by abbreviations for their location: Dhaka, Bangladesh (BGD); Fortaleza, Brazil (BRF); Vellore, India (INV); Bhaktapur, Nepal (NEB); Naushahro Feroze, Pakistan (PKN); Loreto, Peru (PEL); Venda, South Africa (SAV); Haydom, Tanzania (TZH). (Turab *et al*. 2014; Shrestha *et al*. 2014; Lima *et al*. 2014; John *et al*. 2014; Mduma *et al*. 2014; Bessong *et al*. 2014; Yori *et al*. 2014; Ahmed *et al*. 2014).

Enrollment and baseline information has been described elsewhere (MAL‐ED Network Investigators 2014). Briefly, mother–infant dyads were eligible for enrollment in the MAL‐ED study if the infant was less than 17 days of age, a singleton weighing at least 1500 g at birth, without congenital defects or serious illness, born to a mother at least 16 years of age, whose mother was willing to participate in the study and had no plans to move out of the study area for six months (at the time of enrollment). Of the 2145 infants enrolled in the MAL‐ED cohort, analyses were conducted on 1957 infants with complete data to 180 days of age. A total of 188 infants were excluded from analyses here, for the following reasons: 44 were lost to follow‐up, 113 moved out of the study area, 15 died prior to reaching six months of age, 15 had more than 25% missing data from home visits and one infant was enrolled after 17 days of birth.

### Data collection

At enrollment, baseline data on household demographics were collected, which included information on head of household (mother, father, grandparent, other) and maternal characteristics (age, education, parity, pregnancy age). Biweekly nutritional and morbidity surveillance were initiated at the time of enrollment as further explained below. At one and sixth months, maternal depressive symptoms were measured using a Self‐Report Questionnaire (SRQ), and at eight months; maternal reasoning capacities were measured using the Ravens Combined Matrices (RCM) instrument (Murray‐Kolb *et al*. 2014; Pendergast *et al*. 2014).

As described elsewhere (Caulfield *et al*. 2014), nutritional surveillance was conducted through home visits twice weekly, during which the caregiver was queried about the child's consumption in the previous 24 h of breast milk, animal milk, formula, other liquids, water, tea, fruit juice, semi solids and specific solid foods. A second questionnaire, administered monthly, collected more detailed information on non‐breastmilk foods consumed the previous day. Based on the definitions of Labbok and Krasovec, breastfeeding status at each visit was characterized as: *exclusive*, *predominant*, *partial* or *none* (Labbok & Krasovec 1990). Breastfeeding status was defined as EBF in the previous 24 h if the child received only breast milk with the exception of vitamins or medicine. *Predominant* breastfeeding was identified when a child received water or water‐based liquids such as juice or tea in addition to breast milk. If the child received milk‐based liquids, semi‐solid or solid food in addition to breast milk, it was considered *partial* breastfeeding. Finally, a child's breastfeeding status would be categorized as *none*, if there were no consumption of breast milk the day prior to the study visit. For analyses, we further separated *partial* breastfeeding into *partial* breastfeeding with liquids only or *partial* breastfeeding with both liquids and solids to examine the nature of the transition between exclusive and partial breastfeeding status. Days between visits were assumed to have the same status as the preceding visit for the calculation of duration of each breastfeeding practice in days (Henkle *et al*. 2013). Based on our knowledge and site observations, there is very little feeding of expressed breastmilk, and we assume if a child consumed only expressed breast milk that the mother would report this as breastfeeding. Early in our analyses, we observed gaps in EBF, which is the re‐initiation of EBF after at least one visit reported as non‐EBF. Therefore, we quantified the number of these episodes of EBF for each infant over the 6‐month period, the number of days of EBF for each episode of EBF, and considered the number of days of EBF beyond cessation of the first episode as a being the *gain* in EBF. During analyses, we sought to identify maternal and child factors associated with this infant feeding pattern.

In addition to the nutritional surveillance, twice weekly morbidity surveillance was also conducted at the same time, which queried the mother on child's illness symptoms and treatment since the previous visit (number of loose stools, fever, cough, appetite, vomiting, diarrhea, etc.). Detailed information on morbidity definitions and visit frequency have been presented elsewhere (Richard *et al*. 2014).

### Quality control of data

Supervisors at each of the sites performed repeat visits on 5% of households for quality control checks. For the first six months of life, a total of 1731 (average 216 per site) repeat visits were conducted across all of the eight sites. Overall, the percent agreement in reported practices was 90% for EBF, 87% for predominant breastfeeding, 90% for partial breastfeeding and essentially 100% for no breastfeeding. Among mothers who re‐initiated EBF, average agreement among sites was 87% and ranged from 78% in PEL to 100% in BRF for EBF. For predominant feeding, agreement was 85% and ranged from 55% in PKN to 98% in TZH. For partial breastfeeding, agreement was also 90% (51% in PKN to 100% in BRF) and for no breastfeeding, the agreement was 99% across all sites. For diarrhea, agreement was above 93% for all sites (Richard *et al*. 2014).

### Data analyses

For the first aim, we compared three different metrics for estimating the median duration of EBF in the first six months of life, and two summary measures. First, we defined EBF duration for each child as the number of days from birth to the first study visit at which breastfeeding status was not designated EBF; from this, the median duration of EBF collected longitudinally was identified (*EBF_LONG_*). Second, we estimated the proportion of children at each age who were EBF the day before; we randomly chose one visit per infant to make this metric comparable to the other two, and from this estimate, *EBF_DHS_* was identified as the time when 50% of mothers report EBF the day before. We utilized the DHS interpolation method in which age‐stratified proportions of EBF are linearized. The proportion before and after 0.5 (rounded to one decimal) is weighted to estimate the median duration (*EBF_DHS_*) (Rutstein & Rojas 2006; Pullum 2014). Third, we estimated the personal prevalence of EBF, or the proportion of time each infant was EBF during the first 6 months, and estimated the median % days EBF during the first 6 months (*EBF_PREV_*). To estimate the 95% confidence interval (CI) for *EBF_LONG_*, we used survival analysis to first default, and to estimate the 95% CI for *EBF_PREV_* we used a binomial method. Using the data populated from *EBF_DHS_* we also constructed the WHO core indicator (*EBF_WHO_*) which is the proportion of children 0–5.9 months reported as exclusively breastfed (World Health Organization (WHO) 2010), and for comparison, the proportion of children exclusively breastfed using the full longitudinal data (*EBF_TRUE_*).

**Figure 1:**
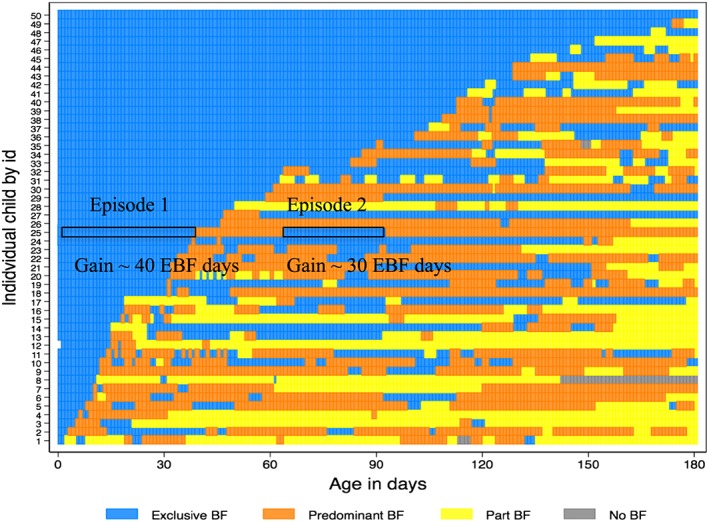
Breastfeeding trajectory plot of 50 children from Loreto, PEL. Each number/row on the y‐axis indicates the pattern of feeding for a child with age in days on x axis. Blue represents exclusive breastfeeding (EBF); orange represents predominant feeding (Predominant BF); yellow represents partial breastfeeding with liquids only (Part BF:liq); brown represents partial breastfeeding with solids (Part BF: sol) and red represents no breastfeeding (No BF). ‘|’ in the sequence indicates when the visit was made. The preceding visit feeding is assumed in the days in between for illustrative purposes. For example, child 25 starts out with exclusive bf, shifts to predominant bf ~day 40, shifts back to exclusive at day 60, which stops at ~day 90. The total gain of EBF days in the first episode of EBF is 40 days and in the second episode, gain is 30 days.

To illustrate individual patterns of feeding over time, breastfeeding trajectory plots were created for each site using 50 randomly selected infants (Fig. Fig. 1 and supplementary Figs 1–7), sorted based on the *EBF_LONG_* metric (Kohler & Brzinsky‐Fay 2005)_._ Each colour in the figure corresponds to their breastfeeding status: blue, EBF; orange, predominant feeding; yellow, partial breastfeeding with liquids only; brown, partial breastfeeding with both liquids and solid; red, no breastfeeding.

To evaluate whether illness episodes and/or low appetite were associated with changes in infant feeding, we evaluated the temporal relations of these factors with the report of non‐EBF, EBF re‐initiation, and with gap length using Chi‐square and *t*‐tests. We also constructed a logistic regression model to identify maternal, child and household characteristics associated with initiating EBF at least three times (i.e. three or more EBF episodes) in the first 180 days. We choose three or more episodes based on frequencies across the sites that would allow comparison, and we posited that this frequency indicated a behaviour. We examined associations with parity (1, 2–4, >5 children), maternal age (years), maternal age at first pregnancy (years), maternal education (0–5, 6–10, 11+ years), maternal reasoning ability and maternal depressive symptoms (average of the two SRQ surveys). When evaluating maternal depressive symptoms, the BRF site was excluded from the model because of measurement concerns (Pendergast *et al*. 2014). Sex was the only child characteristic considered in the logit model. Models were also adjusted for the length of first EBF episode (categorized into 1, 2, 3+ months of EBF), because the longer the duration of the first EBF episode, the lower the likelihood that three or more re‐initiations in the first 180 days would be observed. For household characteristics, we included components of a socio‐economic status scale within the MAL‐ED study called the WAMI index, which includes a composite score based on maternal education, access to improved water and sanitation facilities, assets and monthly income (Psaki *et al*. 2014). For covariates that were collinear, we kept whichever of the covariates provided a more meaningful interpretation. Factors were considered statistically significant at *P* < 0.05 but for these analyses we also included factors marginally significant at *P* < 0.10. Sites were treated as fixed effects in the overall model. Data analyses were conducted using STATA Version 13.1 (StataCorp 2013, College Station, TX).

## Results

Overall, 101, 884 household visits were conducted with 356, 136 days of data in the first 180 days of life collected among 1957 infants. The average number of household visits over the time period across the sites was 53 (IQR: 50, 55) per infant with a median gap of 3 days between visits (IQR: 3, 4) and 96.4% of visits were within 5 days of one another.

Selected demographic characteristics of maternal–infant dyads at each of the eight MAL‐ED sites are shown in Table 1. Overall, the median age at enrollment was 7 days (IQR: 4, 10 days) and the median weight for age Z score at enrollment was −0.7 (IQR: −1.5, −0.1). BRF and SAV had mean maternal BMIs greater than 25.0 kg/m^2^. The mean diarrheal prevalence in the first six months of life ranged from 0.3 days at the BRF site to 24.1 days at the PKN site.

**Table 1:** Descriptive characteristics (% or mean (SD)) of the sample included in the analysis by site

	**BGD**	**BRF**	**INV**	**NEB**	**PEL**	**PKN**	**SAV**	**TZH**	**All sites**
*N*	241	209	234	234	268	263	259	249	1957
Age at enrollment (days)	3.3 (3.0)	9.4 (4.3)	10.1 (3.9)	11.2 (3.5)	5.0 (3.6)	10.4 (4.6)	8.5 (4.7)	7.3 (3.4)	8.1 (4.7)
Weight for age (WAZ) at enrollment	−1.27 (0.9)	−0.20 (1.0)	−1.29 (1.0)	−0.92 (1.0)	−0.60 (0.90)	−1.40 (1.1)	−0.40 (1.0)	−0.14 (0.90)	−0.80 (1.0)
Male infants (%)	49.0	50.7	44.9	53.0	54.1	49.1	50.6	49.4	50.1
First baby (%)	41.1	33.1	34.0	44.1	37.7	21.7	37.1	10.8	32.3
Maternal education < 5 years (%)	62.7	12.9	35.5	25.6	22.8	82.5	2.3	38.2	35.7
Maternal age (y)	24.8 (5.0)	24.0 (5.5)	23.9 (4.2)	26.6 (3.7)	24.3 (6.1)	28.1 (5.9)	26.8 (7.1)	28.6 (6.6)	26.0 (5.9)
Never married (%)	0.0	12.0	0.0	0.0	10.1	0.0	39.4	1.6	8.1
Maternal BMI (kg/m^2^)	22.3 (3.5)	25.6 (4.5)	22.0 (3.9)	25.0 (3.2)	24.7 (3.5)	21.4 (3.7)	26.9 (5.4)	23.0 (3.5)	23.8 (4.4)
Maternal Ravens Matrices raw score	22.8 (10.2)	43.7 (10.4)	44.0 (10.5)	42.0 (13.3)	30.1 (13.1)	25.7 (13.3)	42.7(10.8)	46.5 (8.0)	36.6 (14.5)
Maternal SRQ 16 item score	4.6 (3.5)	a	4.0 (3.7)	2.5 (2.6)	2.4 (2.3)	5.7 (3.5)	3.0 (2.7)	2.7 (2.9)	3.6 (3.3)
Diarrheal prevalence (days) in the first 6 months	6.1 (7.3)	0.3 (1.5)	4.1 (6.4)	7.3 (9.8)	7.8 (9.7)	24.1 (24.6)	0.6 (1.5)	2.0 (3.2)	6.8 (13.2)

MAL‐ED study sites are referred to by abbreviations for their location: Dhaka, Bangladesh (BGD); Fortaleza, Brazil (BRF); Vellore, India (INV); Bhaktapur, Nepal (NEB); Naushahro Feroze, Pakistan (PKN); Loreto, Peru (PEL); Venda, South Africa (SAV); Haydom, Tanzania (TZH).

SRQ data from Brazil not included as it was not possible to factor analyse the data because of lack of variance.

### Dynamics of EBF

Table 2 provides summary information on the breastfeeding metrics. The median duration of EBF to first default (*EBF_LONG_*) was as short as 12 days in PKN to as long as 105 days in BGD. When *EBF_LONG_* was compared to *EBF_DHS_*, large differences were seen for all sites except PKN and SAV. Across all sites, the median duration of *EBF_LONG_* was 33 days (95% CI: 32–36), in contrast to 49 days for *EBF_DHS_*, and 66 days (95% CI: 62–70) for *EBF_PREV_*. There are differences of 4–40 days between *EBF_DHS_* and *EBF_PREV_* (Table 2), with TZH and PEL showing the greatest differences (19 and 40 days, respectively).

**Table 2:** Summary characteristics of EBF metrics and gain in EBF days during the first six months by site

	**BGD**	**BRF**	**INV**	**NEB**	**PEL**	**PKN**	**SAV**	**TZH**	**All sites**
Number of study visits	12,665	11,252	12,272	12,063	14,501	13,252	13,166	12,713	101,884
Visits/child*	53 (51,54)	54 (53,55)	53 (51,54)	52 (50,54)	55 (53,56)	51 (48,53)	52 (49,54)	52 (49,54)	53 (50,55)
Median duration in days (95% CI) *EBF_LONG_*	105 (93–114)	58 (38–62)	83 (71–90)	39 (31–53)	20 (16–27)	12 (11–13)	25 (21–27)	39 (33–44)	33 (32–36)
Median duration in days (linear interpolation) *EBF_DHS_*	140.7	67.8	91.5	84.7	41.7	3.6	13.0	39.2	48.6
Median duration in days (95% CI) *EBF_PREV_*	154 (146–157)	78 (69–92)	107.5 (98–116)	89 (78–105)	82 (67–95)	14 (13–19)	32 (29–37)	58 (51–62)	66 (62–70)
*EBF_WHO_* (%)	71.3	47.3	56.8	46.1	49.2	13.3	21.6	29.3	41.3
*EBF_TRUE_* %	10.4	4.8	0.9	0.9	1.1	0.0	0.0	0.0	2.2
One episode of EBF only (%)	29.8 (*n* = 72)	41.1 (*n* = 86)	25.6 (*n* = 60)	19.2 (*n* = 45)	16.8 (*n* = 45)	66.5 (*n* = 175)	46.3 (*n* = 120)	39.0 (*n* = 97)	35.8 (*n* = 700)
Episodes of EBF *	2 (1,3)	2 (1,3)	2 (1,4)	3 (2,4)	3 (2,5)	1 (1,2)	2 (1,3)	2 (1,2)	2 (1,3)
Gain in EBF days *									
Second episode (*n* = 1250)	21 (7,52)	21 (4,30)	10 (4,27)	8 (4,21)	7 (4,19)	4 (3,14)	4 (3,9)	6 (4,17)	7(4,25)
Third episode (*n* = 756)	9 (4,21)	22 (7,30)	6 (3,14)	7 (4,16)	6 (3,14)	4 (3,7)	6 (4,14)	7 (3,15)	7 (3,15)
Fourth episode (*n* = 464)	7 (4,17)	24 (7,29)	7 (4, 14)	7 (3,24)	7 (3,14)	4 (3,7)	4 (3,7)	5 (3,10)	7 (3,14)
Fifth episode (*n* = 262)	4 (3,9)	12 (3,21)	6 (4,9)	7 (3,16)	6 (3,12)	4 (3,9)	4 (2,11)	4 (3,14)	6 (3,11)

* Median (interquartile range) reported for these variables.

MAL‐ED study sites are referred to by abbreviations for their location: Dhaka, Bangladesh (BGD); Fortaleza, Brazil (BRF); Vellore, India (INV); Bhaktapur, Nepal (NEB); Naushahro Feroze, Pakistan (PKN); Loreto, Peru (PEL); Venda, South Africa (SAV); Haydom, Tanzania (TZH).

*EBF_LONG_* is defined as the EBF duration for each child as the number of days from birth to the first report of non‐EBF; *EBF_DHS_* is interpolated from data on the proportion of children at each age who were EBF the day prior; *EBF_PREV_* is the proportion of days each infant was EBF during the first 6 months. If EBF duration was punctuated with a period of non EBF, that duration would be considered as an episode of EBF and if the mother re‐initiates EBF, the number of days she subsequently reports EBF is measured as the gain in EBF, and these are estimated each time this is observed over the six‐month period.

According to the WHO core indicator (*EBF_WHO_*), between 13.3% (PKN) and 71.3% (BGD) children 0–5.9 months were EBF; however, based on the longitudinal data (*EBF_TRUE_*), 0% (PKN, SAV, TZH) to 10.4% (BGD) were truly EBF at 6 months.

Overall 35.8% of the mothers initiated EBF only once, varying from 16.8% to 66.5% across sites, and the median number of episodes of EBF ranged from 1 (PKN) to 3 (PEL). The breastfeeding trajectory plot for PEL (Fig. 1) illustrates the multiple episodes of EBF experienced by infants during the first 180 days. As an example, the breastfeeding trajectory for infant # 25 includes two episodes of EBF—a first episode lasting 40 days, and with the second episode, a *gain* of 30 days of EBF.

**Figure 2:**
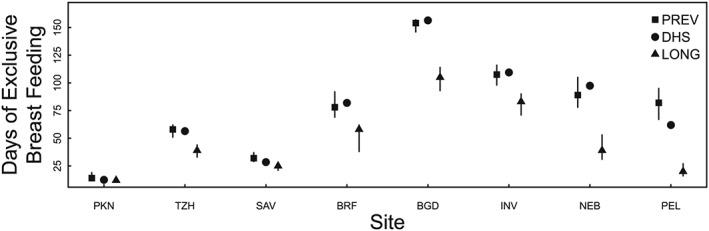
Three metrics for EBF median duration are arranged by median re‐initiation of EBF, from low (PKN) to high (PEL): *EBF_LONG_* is the longitudinal method (from birth to first reported non‐breastmilk substance), *EBF_DHS_* is calculated using the DHS interpolation method, and *EBF_PREV_* is the personal prevalence. MAL‐ED study sites are referred to by abbreviations for their location: Dhaka, Bangladesh (BGD); Fortaleza, Brazil (BRF); Vellore, India (INV); Bhaktapur, Nepal (NEB); Naushahro Feroze, Pakistan (PKN); Loreto, Peru (PEL); Venda, South Africa (SAV); Haydom, Tanzania (TZH).

**Figure 3:**
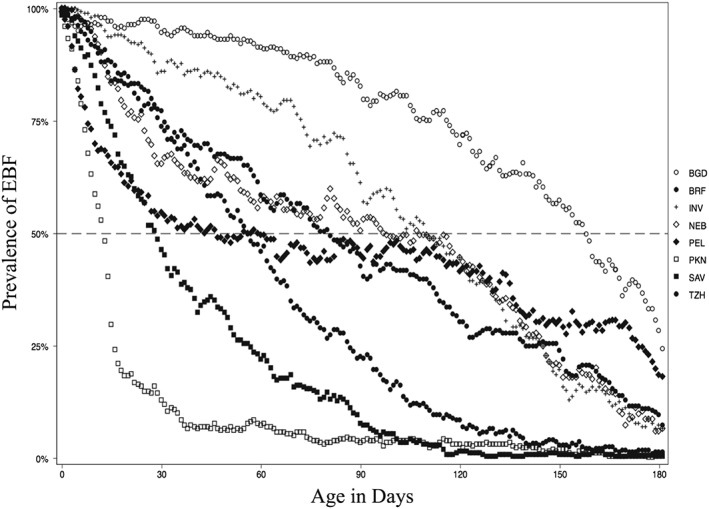
Prevalence of EBF in the previous day by MAL‐ED sites. Each site has 180 markers representing the prevalence of EBF for the previous day (from day 1 to 180 of child's life).

Fig. 2 summarizes the three EBF duration metrics across the sites by median re‐initiation of EBF, from low (PKN) to high (PEL). Sites where a higher proportion of women initiated only once had overlapping estimates for EBF duration across the three methods. With increasing EBF re‐initiations, the metrics diverged, with greater agreement remaining between *EBF_DHS_* and *EBF_PREV_*. Shown in Fig. 3, is the daily EBF prevalence in the first 180 days for each of the MAL‐ED sites. The curve with the drastic decline in the first month followed by gradual decline of EBF prevalence leads to a lesser degree of overestimation as in the case of PKN. In contrast, in PEL where the highest number of EBF re‐initiations were observed had a slower rate of decline between month one and four, where the prevalence hovers around the median, leading to large discrepancies among the various metrics.

Presented in Table 2 are the *gains* in EBF days associated with two to five EBF episodes within the first six months. If a child had three episodes of EBF (i.e. mother re‐initiated twice more) across the first 180 days, the durations for the second and third episodes are included in the summary as second and third episode, respectively. Not surprisingly, the number of mothers that re‐initiate declined with each additional episode of EBF. In the second episode of EBF, infants on average gain 21 EBF days in BGD and BRF, 7–10 days in PEL, INV and NEB and 4–6 days in TZH, SAV and PKN. With the third episode of EBF, the gain for BRF was 21 days, but for the other sites, it was on the order of 4–9 days. The greatest number of EBF episodes was observed in the PEL site, where one infant experienced 16 episodes of EBF.

The distribution of the re‐initiation episodes in the first 6 months varied across sites as shown in Table 3. This is directly related to the duration of the first EBF episode. In PKN, SAV and TZH, the median age at which the highest number of episodes observed was between 47 and 66 days, whereas in NEB, INV and BGD, it was 91 and 117 days. In PEL and BRF, the distributions of episodes were constant throughout the first six months.

**Table 3:** Characteristics of re‐initiation episodes: distribution, gaps, morbidity and feeding profiles

	**BGD**	**BRF**	**INV**	**NEB**	**PEL**	**PKN**	**SAV**	**TZH**	**All sites**
Distribution of EBF episodes in 180 days*	117 (82,149)	91 (54,122)	102 (66,137)	92 (59,128)	97 (55,141)	55 (28,100)	47 (30,66)	66 (38,101)	89 (49,128)
Duration of gaps between EBF episode*									
First gap	4 (3,7)	2 (2,8)	7 (4,11)	7 (4,21)	8 (4,25)	14 (4,60)	7 (3,15)	7 (4,14)	7 (3,15)
Second gap	7 (3,14)	2 (2,7)	7 (4,10)	7 (3,16)	8 (4,21)	6 (4,21)	8 (4,15)	6 (4,14)	7 (3,14)
Third gap	7 (4,11)	2 (2,4)	7 (4,18)	7 (4,13)	10 (4,18)	6 (3,12)	7 (3,14)	7 (4,14)	7 (4,15)
Fourth gap	5 (4,16)	4 (2,7)	7 (4,16)	4 (3,11)	7 (4,14)	3 (2,8)	6 (2,11)	5 (4,14)	7 (3,14)
EBF episodes preceded by diarrhea (%) (*n* = 314)	16.0	0.5	9.5	11.5	11.3	28.8	1.6	5.2	10.4
EBF gain in days (*P* value)	0.8287	a	0.9058	0.9779	0.6605	0.9513	a	0.1766	0.9939
When preceded by diarrhea*	14 (4,35)	b	4 (4,10)	7 (4,21)	7 (3,14)	4 (3, 10)	4 (4, 6)	4 (3, 7)	7 (3,15)
When not preceded by diarrhea*	10 (4,28)	21 (4,29)	7 (4,14)	7 (4,20)	7 (3,14)	4 (3,10)	4 (3,8)	6 (3,14)	7 (3,18)
Type of non‐EBF prior to re‐initiation of EBF (%)									
Predominant BF	57.7	49.3	40.9	43.0	74.2	66.7	81.6	27.1	56.2
Partial BF without solids	42.0	46.7	58.0	55.7	25.3	20.1	16.3	72.2	42.0
Partial BF with solids	0.3	0.9	1.1	1.0	0.4	0.0	1.1	0.6	0.7
No BF	0.0	3.1	0.0	0.2	0.0	13.2	1.1	0.0	1.1
EBF gain									
(*P* value)	0.0000	0.5562	0.0000	0.0834	0.3214	0.0306	0.7766	0.0000	0.0000
When preceded by predominant BF*	17 (7,43)	18 (3,30)	10 (4,27)	10 (4,21)	7 (4,14)	5 (3,13)	4 (3,8)	14 (6,25)	7(4,21)
When preceded by Partial BF without solids*	5 (3,14)	24 (7,30)	6 (3,11)	7 (3,14)	4 (3,14)	4 (3,7)	6 (3,8)	4 (3,8)	6 (3.14)

* Distribution, gaps and gain in EBF are reported in median days (IQR).

MAL‐ED study sites are referred to by abbreviations for their location: Dhaka, Bangladesh (BGD); Fortaleza, Brazil (BRF); Vellore, India (INV); Bhaktapur, Nepal (NEB); Naushahro Feroze, Pakistan (PKN); Loreto, Peru (PEL); Venda, South Africa (SAV); Haydom, Tanzania (TZH).

a T‐test not performed when observations were less than five (*n* < 5).

b Only one observation.

Of 3, 024 episodes of EBF, only 10.4% had diarrhea in the 7 days prior to the re‐initiation of EBF. Of these, 43% of the EBF gains were <5 days, 14% lasted 5–9 days and another 15% lasted 10–20 days of EBF. Average EBF gains in each site were not significantly different when compared to re‐initiation episodes that were not preceded by a diarrhea episode. Other illness symptoms such as fever and vomiting were present in the week prior for <1% of the EBF episodes.

Cessation of EBF episodes with maternal report of infant's appetite (up to 7 days prior to the cessation) was examined and only 3% of the times mother reported poor appetite prior to cessation. In addition, the duration and type of gaps were explored (days of non‐EBF days between episodes and type of food fed in those days) between EBF episodes and found to be constant across episodes of re‐initiation within sites. The median number of days between any two episodes within the first four episodes of EBF was 7 days (IQR: 3–14 days). Between the first and second episode, the smallest gap was observed in BRF, where the median gap was 2 days (IQR: 3–7) while the largest gap was seen in PKN where mothers took a median of 14 days to re‐initiate the second episode (IQR: 4–60 days). This trend was consistent across re‐initiation for subsequent episodes for all sites except PKN.

For the first five re‐initiations of EBF, there was a consistent trend in that 57% of the shifts were switching from predominant feeding to EBF, 42% were switching from partial feeding without solids, a small percentage of episodes were switching from partial feeding with solids (<1%) and from no breastfeeding to EBF (<2%). However, within sites, the shifts to EBF from predominant BF was highest in SAV with 81.6% and lowest in TZH with 27.1% and was consistent for subsequent re‐initiations. Differences in EBF gain were examined when switching from predominant BF vs. partial BF without solids to EBF. Across eight sites, switching from predominant BF to EBF, a child on average had an EBF gain of 16.6 days (SD: 22.1) whereas switching from partial BF without solids to EBF resulted in a significantly lower gain of 12.4 days (SD: 17.6) at *p* < 0.0000. However, within sites the EBF gain when switching from predominant BF or partial BF without solids was not significantly different in BRF, PEL, NEB or SAV.

### Maternal and household characteristics and EBF re‐initiation

From these analyses, we concluded that re‐initiation of EBF is unrelated to caregiver reports of concurrent illness or poor appetite. Given these results, we sought to understand the maternal, child and household factors affecting multiple episodes of EBF re‐initiation.

Shown in Table 4 is a set of logistic models, identifying factors associated with having 3+ EBF episodes. There were two variables that were collinear: (1) parity and age of the mother, and (2) maternal education and reasoning capacity. In both these cases, we kept the second variable for meaningful interpretation. Two factors were identified across eights sites after adjusting for socio‐economic factors. The shorter duration of the first EBF episode (*EBF_LONG_*) was inversely associated with the higher likelihood of three or more EBF episodes in the first 180 days. Higher maternal reasoning capacity was associated with greater odds of three or more EBF episodes, particularly in BGD and NEB. We observed site‐specific differences in the covariates, notably in four sites. In BGD, older mothers were likely to re‐initiate, whereas having more assets and a higher monthly income increased the likelihood of re‐initiation in TZH. In PEL, self‐reported symptoms of depression were associated with increased re‐initiation. In SAV, access to improved water source and sanitation facility decreased the likelihood of re‐initiation.

**Table 4:** Factors associated with having three or more+ episodes of EBF in pooled analyses OR [95% CI]

	BGD	BRF	INV	NEB	PEL	PKN	SAV	TZH	All sites
Observations	214	189	225	224	250	248	113	159	1437
Mothers age (per 5 year)	1.402*	1.099	0.883	0.890	0.817+	0.921	1.206	0.977	0.988
	[1.023,1.923]	[0.819,1.474]	[0.633,1.231]	[0.604,1.312]	[0.653,1.021]	[0.670,1.267]	[0.876,1.659]	[0.724,1.318]	[0.886,1.100]
Duration of EBF first episode									
>3 month	Reference								
2 month	4.732*	1.816	1.885+	1.750	1.962	5.200*	2.224	1.955	2.418*
	[1.993,11.23]	[0.816,4.043]	[0.953,3.728]	[0.824,3.715]	[0.837,4.600]	[1.583,17.08]	[0.425,11.62]	[0.703,5.442]	[1.730,3.380]
1 month	4.927*	2.255*	4.979*	2.178*	2.170*	1	2.512	3.449*	2.818*
	[2.058,11.80]	[1.096,4.641]	[2.205,11.24]	[1.152,4.117]	[1.175,4.006]	[1,1]	[0.556,11.35]	[1.337,8.899]	[2.088,3.803]
Maternal reasoning capacity (per 10 units)	1.363*	0.993	1.094	1.201+	1.052	1.171	0.897	1.534	1.142*
	[1.019,1.824]	[0.730,1.352]	[0.828,1.445]	[0.968,1.490]	[0.849,1.303]	[0.893,1.535]	[0.603,1.335]	[0.911,2.584]	[1.032,1.263]
Maternal depressive symptoms (per 2 units)	1.005	a	0.991	1.105	1.335*	1.094	0.836	0.864	1.046
	[0.838,1.204]		[0.839,1.171]	[0.888,1.375]	[1.011,1.765]	[0.870,1.377]	[0.545,1.282]	[0.622,1.201]	[0.964,1.135]
Assets	0.959	1.084	1.006	1.127	0.991	0.983	0.941	1.206+	1.050
	[0.799,1.150]	[0.774,1.519]	[0.852,1.187]	[0.940,1.351]	[0.822,1.194]	[0.803,1.203]	[0.711,1.247]	[0.965,1.508]	[0.979,1.125]
Improved sanitation and water access score	1	0.712	1.101	1	0.937	1.169	0.764*	0.979	0.974
	[1,1]	[0.378,1.341]	[0.951,1.274]	[1,1]	[0.818,1.074]	[0.898,1.522]	[0.589,0.991]	[0.839,1.143]	[0.906,1.046]
Monthly income in USD(per $1)	1.000	1.000	1.000	0.999	1.002	1.000	1.000	1.014*	1.000
	[0.997,1.003]	[0.998,1.003]	[0.994,1.006]	[0.997,1.001]	[0.998,1.006]	[0.996,1.003]	[0.998,1.002]	[1.002,1.026]	[0.999,1.001]

+ *p* < 0.10.

* *p* < 0.05.

a SRQ data from Brazil not included as it was not possible to factor analyse the data because of lack of variance.

MAL‐ED study sites are referred to by abbreviations for their location: Dhaka, Bangladesh (BGD); Fortaleza, Brazil (BRF); Vellore, India (INV); Bhaktapur, Nepal (NEB); Naushahro Feroze, Pakistan (PKN); Loreto, Peru (PEL); Venda, South Africa (SAV); Haydom, Tanzania (TZH).

## Discussion

Across the eight sites of the MAL‐ED study, we demonstrate differences in the median duration of EBF based on three commonly used metrics in the literature. Overall, the median duration of EBF using the longitudinal indicator was 33 (95% CI: 32–36) days, compared to 49 days following DHS methodology and 66 (95% CI: 62–70) days with personal prevalence. Other studies, both in the developed and developing world have illustrated differences when EBF is measured via the DHS method vs. first default method (Zohoori *et al*. 1993; Aarts *et al*. 2000; Cattaneo *et al*. 2000; Engebretsen *et al*. 2007; Agampodi, T. C. Agampodi and de Silva 2009; Agampodi & Agampodi 2009; Agampodi *et al*. 2011). A few studies have also described diverse patterns through which infants pass between breastfeeding categories, and Zoohori *et al.* posited this as a reason for discrepancies across metrics (Zohoori *et al*. 1993; Ssenyonga *et al*. 2004; Engebretsen *et al*. 2007). However, our report is the first study to quantify the reasons for these differences in the EBF estimates. Our analyses indicate that the magnitude of the differences in the metrics varies across the sites and are related (1) inversely to the time from birth to the first time EBF (*EBF_LONG_*), and, (2) directly to the frequency with which women stop and re‐start EBF and the gain in days associated with each new EBF episode. With increasing frequency of re‐initiations, EBF*_PREV_* and EBF*_DHS_* diverge from EBF*_LONG_* but the median duration derived from cross‐sectional data, EBF*_DHS_*, does approximate the median duration based on the longitudinal personal prevalence measure EBF*_PREV_*. We were able to evaluate these differences because of the intense follow up of infants documenting breastfeeding practices every few days for the first six months of life. Further, quality control visits showed high overall agreement between the supervisor and fieldworker (>90%) and importantly, agreement was also high (87%) among those mothers who re‐initiated EBF multiple times.

Our results involving four sites in South Asia, two in Sub‐Saharan Africa and two in Latin America indicate that many infants experience multiple episodes of EBF during the first 180 days of life. Across sites, 64.2% of the women re‐initiated EBF after cessation. The majority of re‐initiation episodes occurred when the infants were 2–3 months of age and infants gained an average of 7–23 EBF days. Given their age, such gains in EBF are potentially important to the health of the baby. Importantly, neither stopping nor re‐starting EBF was associated with diarrhea, other reported illness or reported appetite. Although 56% of gaps in EBF involved the introduction of waters and low energy fruit juices, 43% did involve the feeding of other milks or milks and solids. The introduction of waters may not affect breastmilk production or consumption but it is associated with increased risk of morbidity and mortality and should be discouraged (Popkin *et al*. 1990; Arifeen *et al*. 2001). The temporary switch to partial or no breastfeeding is more problematic as it may diminish breastmilk production and mothers may need programmatic support to increase their milk supply to support continued EBF. As documented previously, BGD, BRF, PEL and INV sites have established programmes promoting EBF, whereas PKN, TZH and SAV have few programmes in place (Patil *et al*. 2015).

Understanding what factors lead to stopping EBF or to re‐initiation may lead to the development of materials and/or messages for counselling programmes aimed at increasing the duration of EBF across settings. We did not ask women why they made various infant feeding decisions, and this is a limitation for these analyses. We also did not collect information on mode of breast milk consumption (breastfed or expressed and bottle‐fed), and assume women report both as breastfeeding. Further, we were unable to estimate the confidence interval through boot strapping techniques for the EBF*_DHS_* method because this was an aggregate level measure; it would likely be statistically inefficient (Dr. T Pullam 2015, personal communication, 21 October). Although, there are other ways to estimate the median and CI (e.g. Bonnett and Price, 2002), the median estimated from these methods does not align with the linearly interpolated method used for the DHS (Bonett & Price 2002).

We sought to identify infant, maternal and household factors associated with more frequent stopping and re‐starting EBF. Greater maternal reasoning ability and shorter duration of EBF of less than <2 months increased the likelihood of re‐initiating, and within some sites older mothers, and those living in households with more assets and higher monthly income were more likely to re‐initiate. Thus, in general, it appears that older, more experienced and those with greater reasoning abilities and more assets were more likely to report gaps in EBF.

The current target set by the World Health Assembly (WHA) is to reach 50% EBF among infants 0–5 months of age across the world through a variety of interventions, identified at the community, hospital and national level (World Health Organization (WHO) 2014; Rollins *et al*. 2016). In this report, two sites (BGD [71.3%] and INV [56.8%]) reach that goal. The data also suggest that only 1–10% of infants were actually EBF to 6 months, and the median durations of EBF using DHS method were 92–141 days, and the median prevalence of days EBF were 108–154 days. Because the response data are the same for both the WHO core indicator and the DHS duration measure, and the median duration is arguably more informative for tracking progress and approximates the median personal prevalence (a true longitudinal measure of EBF), it is not clear why the WHO core indicator would be preferable over the prior DHS metric. Based on our findings, we speculate that survey instruments could include questions such as ‘how often have you stopped and re‐initiated EBF (never, rarely, sometimes, often)’ to adjust the median DHS method to approximate the personal prevalence measure. Some researchers have suggested asking directly about ‘EBF since birth’ and a longer recall period (<1 week) as other methods to accurately capture the duration of EBF (Huttly *et al*. 1990; Aarts *et al*. 2000; Bland *et al*. 2003; Engebretsen *et al*. 2007). Further research is needed to establish the sensitivity of these metrics to detect change in breastfeeding practices. Multiple indicators should be used to evaluate programmes, given the heterogeneity of EBF patterns in the first six months of life. Programme planners should also investigate the extent to which infants experience multiple episodes of EBF, and to encourage and support EBF re‐initiation as they work in general to extend the duration of the first episode of EBF to six months.

## Source of funding

The Etiology, Risk Factors and Interactions of Enteric Infections and Malnutrition and the Consequences for Child Health and Development Project (MAL‐ED) is carried out as a collaborative project supported by the Bill & Melinda Gates Foundation, the Foundation for the NIH and the National Institutes of Health/Fogarty International Center. Ramya Ambikapathi received the Kruse Family Publications Award and is funded by the Integrative Graduate Education and Research Traineeship (IGERT Award 1069213), a JHU Environment, Energy, Sustainability and Health Institute (E^2^SHI) fellowship and JHSPH Center of Global Health.

## Conflicts interest

The authors declare that they have no conflict of interest.

## Contributions

The concept for the analysis and paper were conceived by RA and MK and focused on feeding practices at the Peru site. The results were presented in part at Experimental Biology 16, April 27, 2014 in San Diego, CA. It was then expanded to include the other seven of the eight MALED sites. The analysis was conducted by RA, and the analysis and interpretation of the results involved RA, MK, LEC, BJJM, LMK, GL and DL. CM, CP, BL, AT, MMI, MU, AB and MPO led collection of the nutrition data at each of the sites, and contributed to the interpretation of results and the manuscript. All authors contributed to the final interpretation of the data, and writing of the final manuscript. All authors have approved the content of the manuscript.

## Supporting information


**Figure S1**. Breastfeeding trajectory plot of 50 children from Dhaka, BGD.
**Figure S2**. Breastfeeding trajectory plot of 50 children from Naushahro Feroze, PKN.
**Figure S3**. Breastfeeding trajectory plot of 50 children from Bhaktapur, NEB.
**Figure S4**. Breastfeeding trajectory plot of 50 children from Vellore, INV.
**Figure S5**. Breastfeeding trajectory plot of 50 children from Fortaleza, BRF.
**Figure S6**. Breastfeeding trajectory plot of 50 children from Venda, SAV.
**Figure S7**. Breastfeeding trajectory plot of 50 children from Haydom, TZH.

Supporting info item
