# Supplementary material for: How multiple episodes of exclusive breastfeeding impact estimates of exclusive breastfeeding duration: report from the eight‐site MAL‐ED birth cohort study
Source: Matern Child Nutr. 2016 Aug 8;12(4):740–56. doi: 10.1111/mcn.12352 (PMC5095788; doi:10.1111/mcn.12352)
Supplement: Supplementary file 1 — Supporting info item [file MCN-12-740-s001.doc]

**Supplementary figures:**

Supplemental Figure 1: Breastfeeding Trajectory plot of 50 children from Dhaka, BGD. **
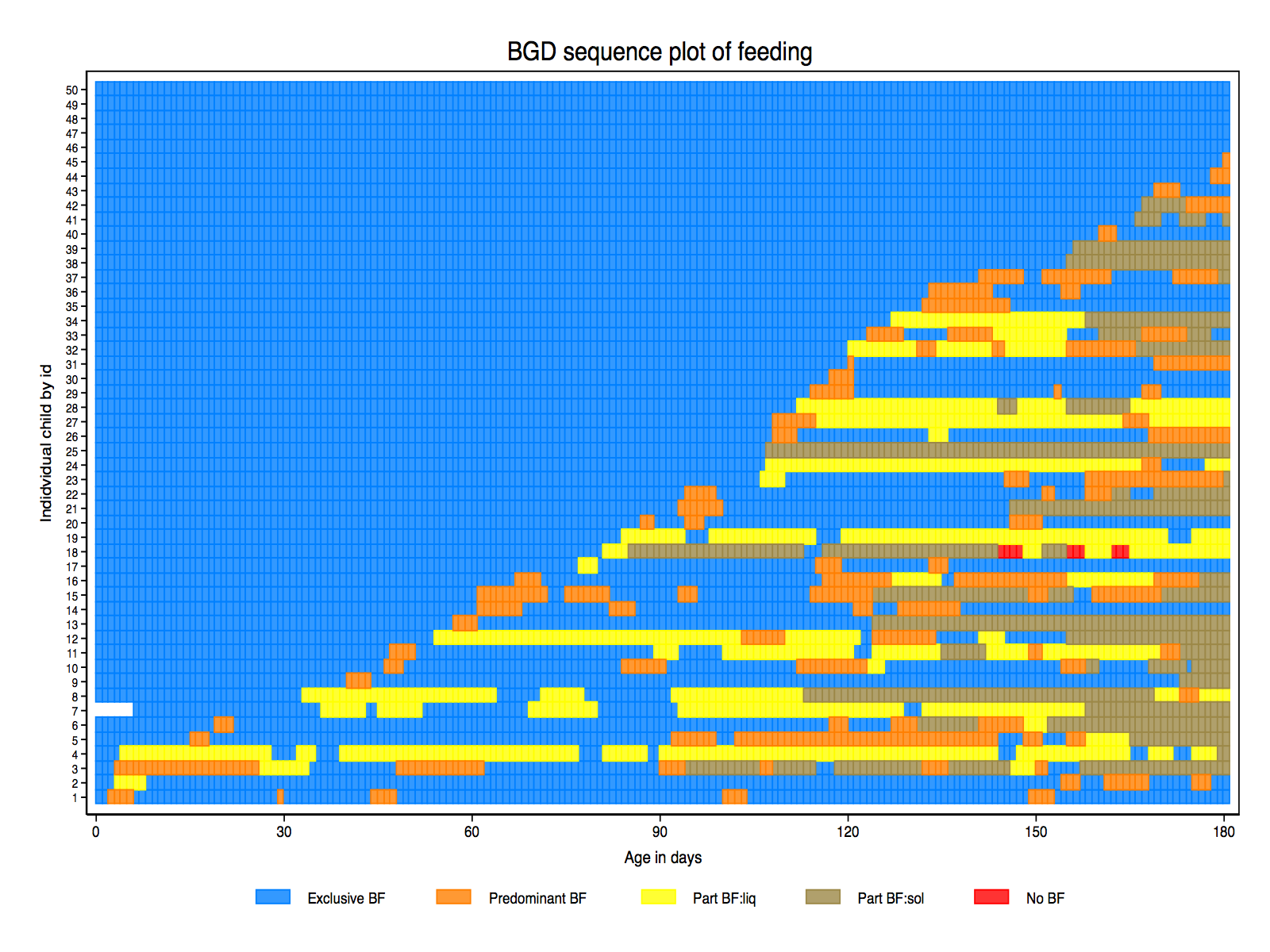
**

Supplemental Figure 2: Breastfeeding Trajectory plot of 50 children from Naushahro Feroze, PKN.

**
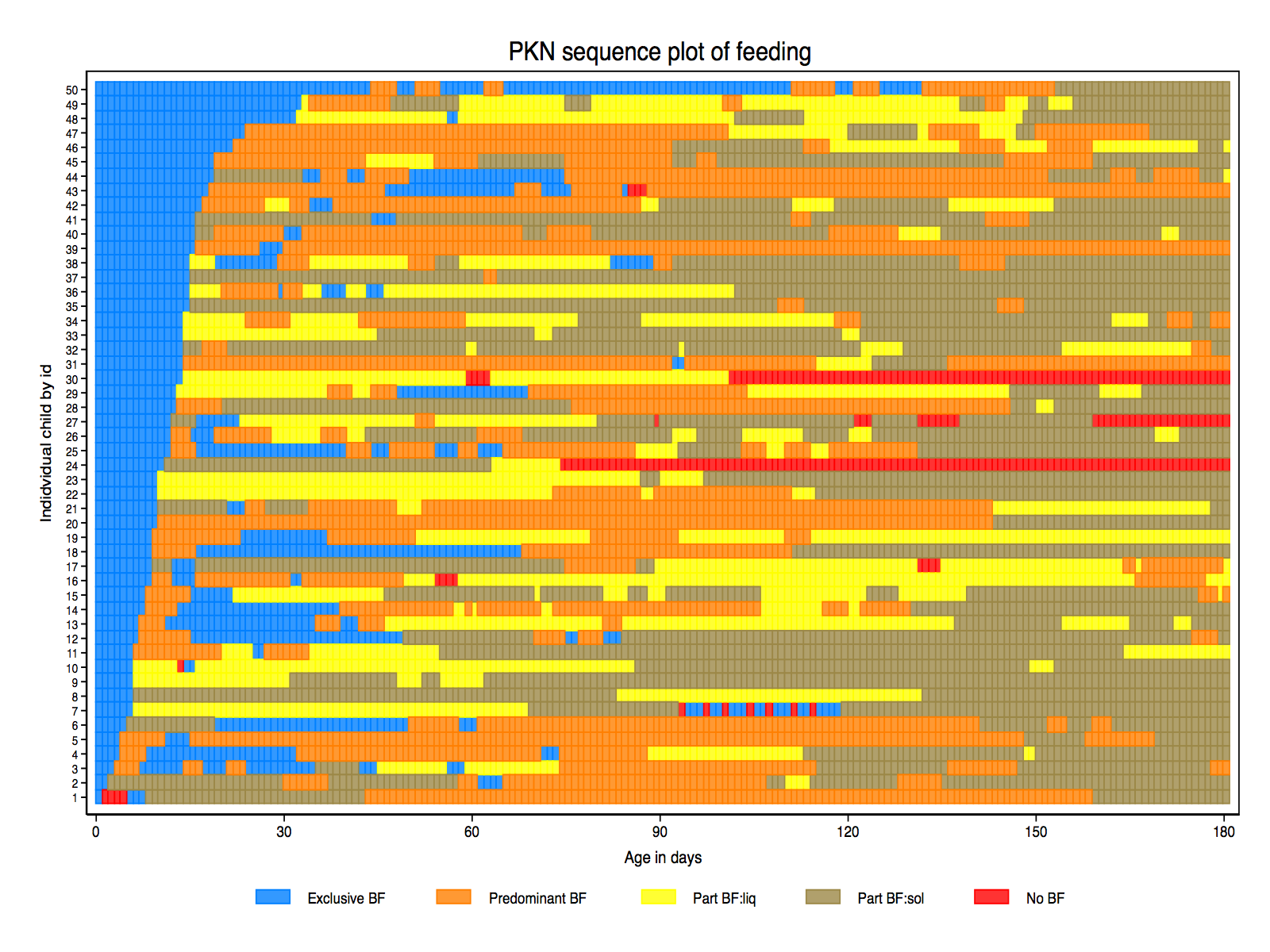
**

Supplemental Figure 3: Breastfeeding Trajectory plot of 50 children from Bhaktapur, NEB. **
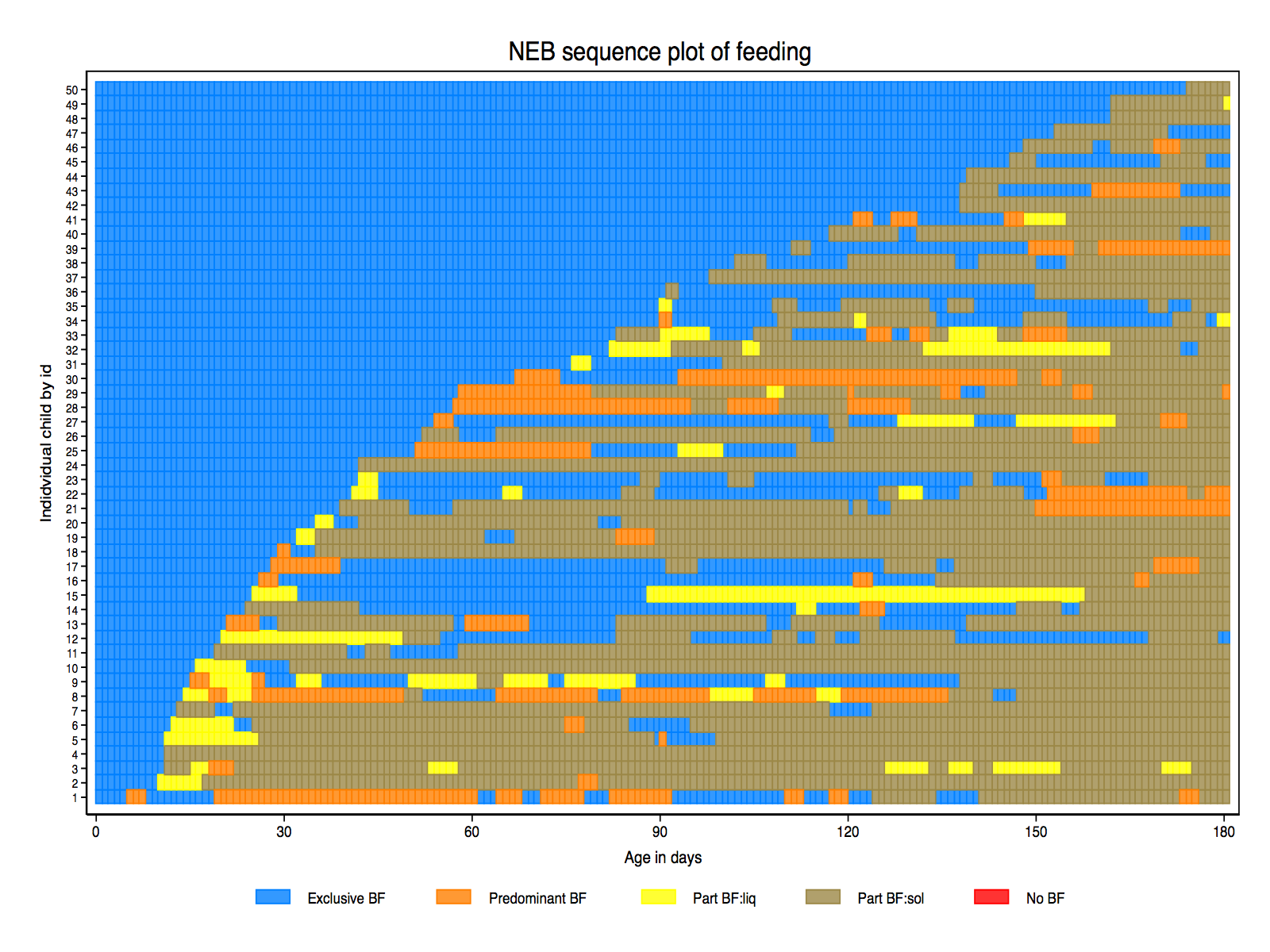
**

Supplemental Figure 4: Breastfeeding Trajectory plot of 50 children from Vellore, INV.

**
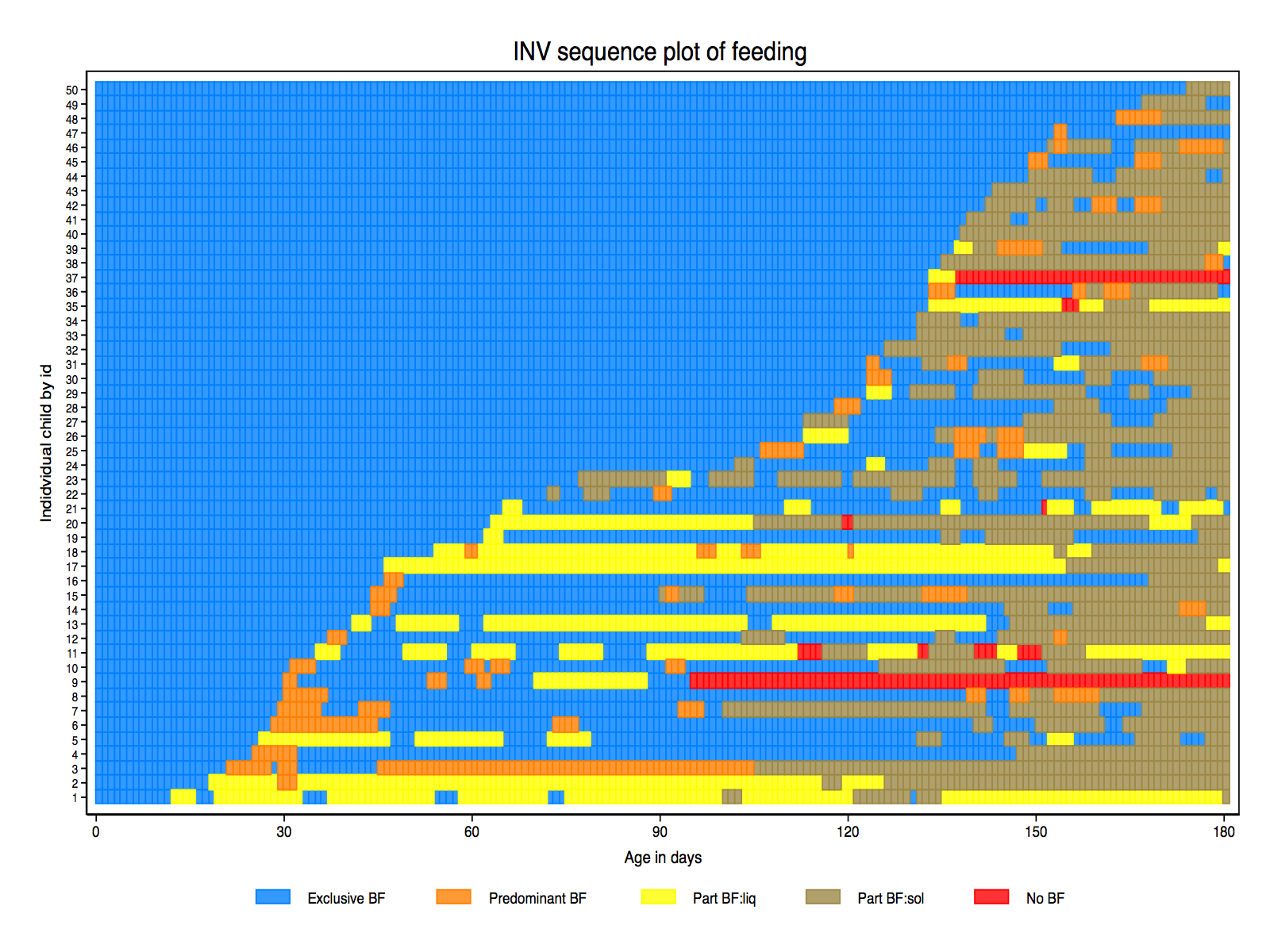
**

Supplemental Figure 5: Breastfeeding Trajectory plot of 50 children from Fortaleza, BRF.

**
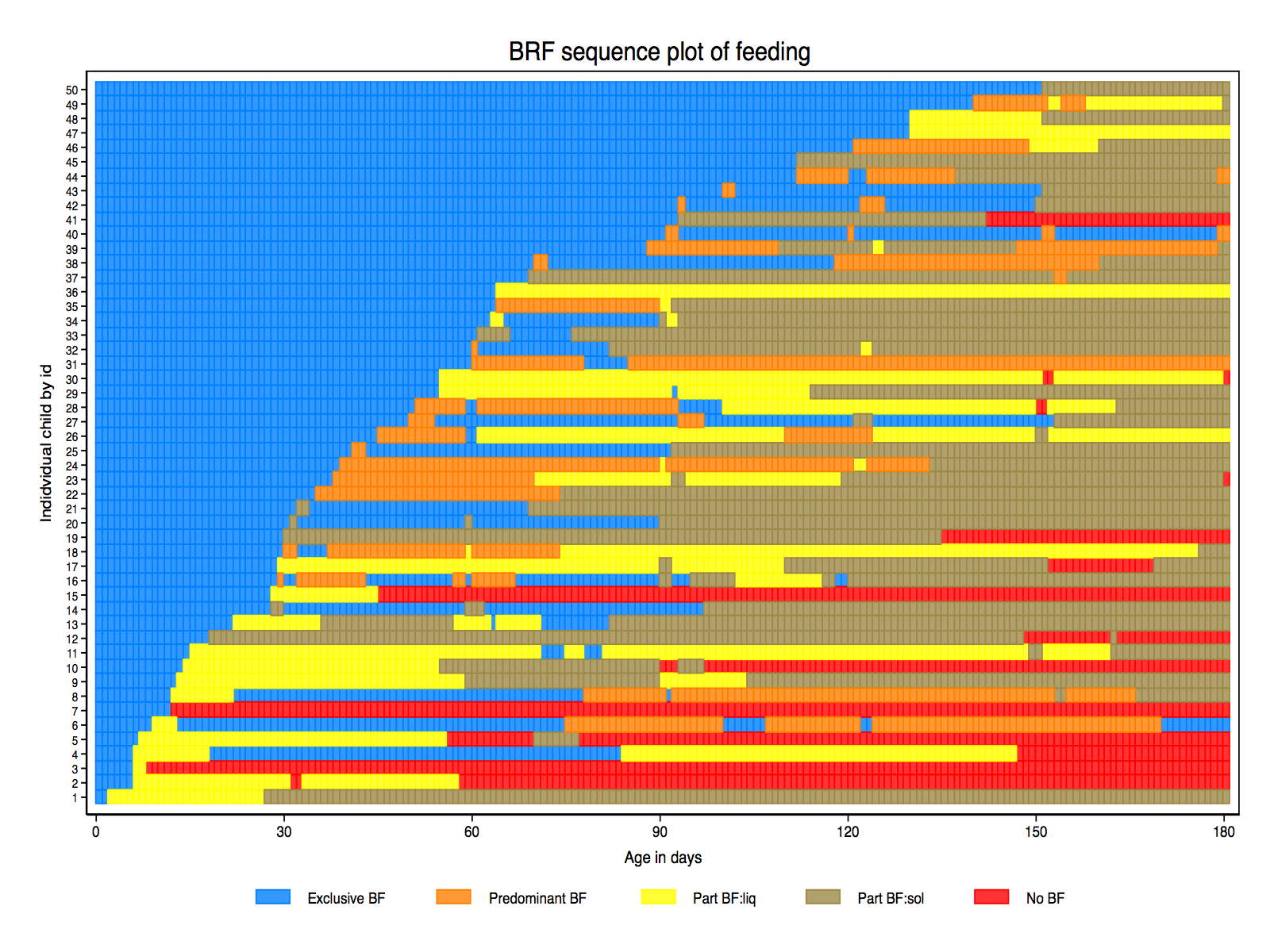
**

Supplemental Figure 6: Breastfeeding Trajectory plot of 50 children from Venda, SAV.

**
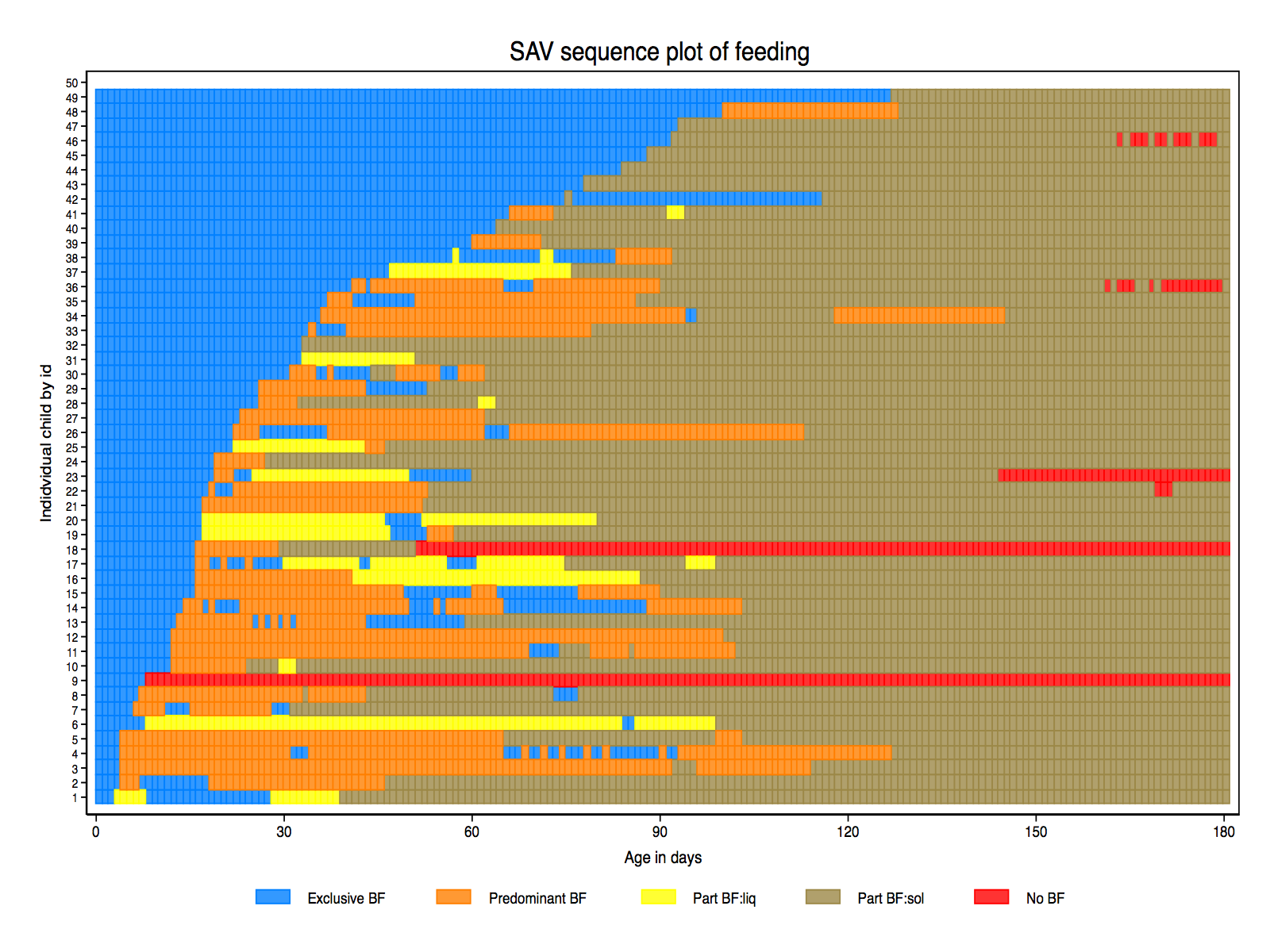
**

Supplemental Figure 7: Breastfeeding Trajectory plot of 50 children from Haydom, TZH.

**
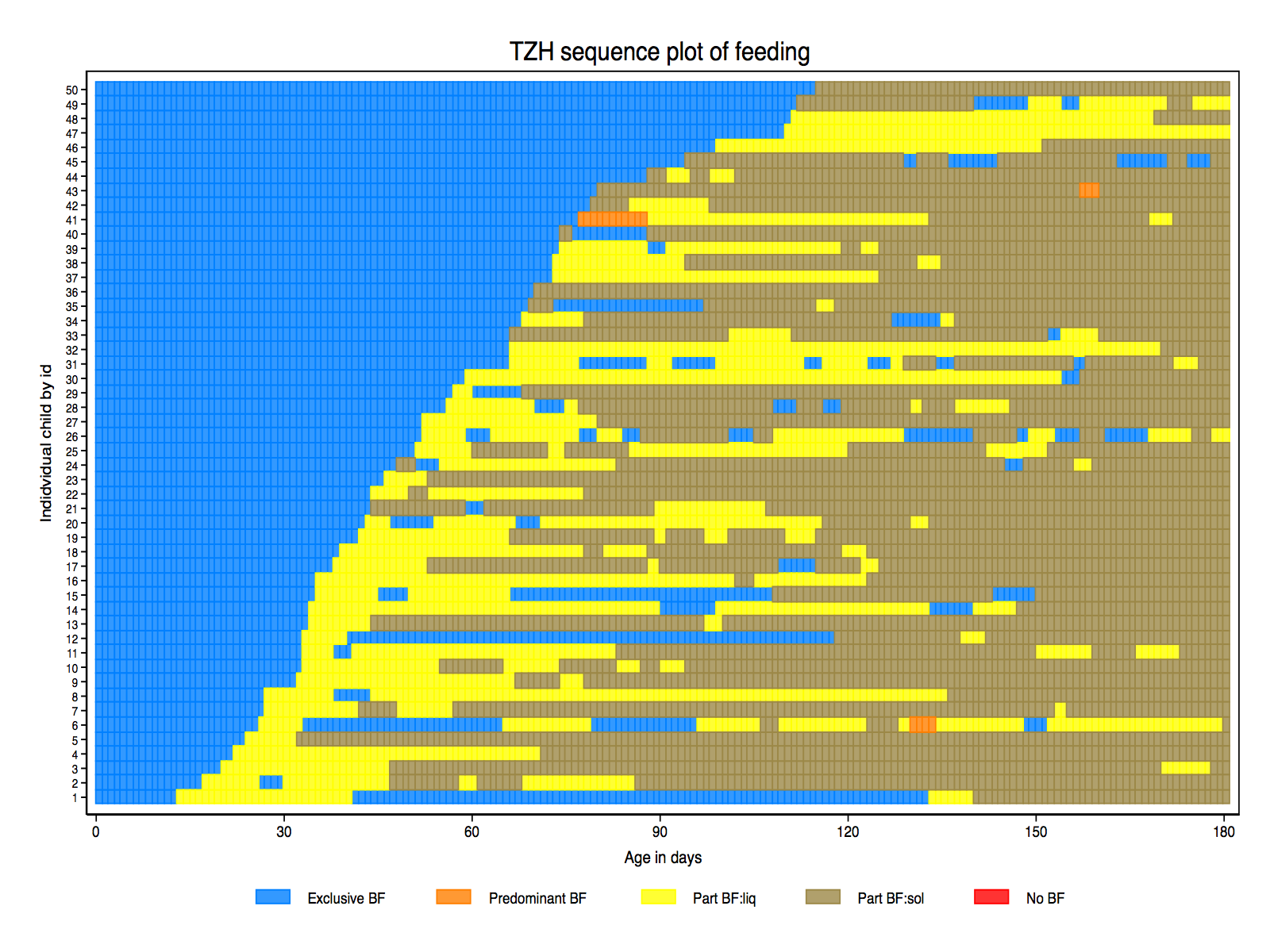
**
